# Pennsylvania

**Published:** 1896-08

**Authors:** 


*Pennsylvania.* Through the daily press, rabies among the dogs and horses of Altoona, Pa., has been reported.

This disease continues to prevail also in a circumscribed district in Philadelphia. Fears are entertained at Cloverdell Farm, near Philadelphia, the home of so many of the most famous sires and brood-mares, as well as valuable kennels of collies and St. Bernards, as to the outcome of a suspected case of rabies in one of their own dogs, and which fled from section to section of this great breeding establishment before he was destroyed. Dr. E. Mayhew Michener, of North Wales, Pa., is making a thorough investigation of the matter.

Dr. E. Mayhew Michener, of North Wales, has destroyed, under the direction of the State Veterinary Sanitary Board, one hundred head of cattle in the eastern section of Pennsylvania, adjacent to Philadelphia. He estimates that 5 to 6 per cent. of the cattle in Montgomery, Bucks, Chester, and Delaware counties are affected with tuberculosis.

Another herd at Doylestown has had eight out of nine Guernseys condemned and destroyed.

The eight cattle condemned and quarantined at Atherton, Westmoreland County, are reported as improved in general appearance.

A herd near North Wales, numbering seventeen, mostly Jerseys, revealed thirteen tuberculous ones.

On July 16th a number were destroyed at Columbia.

Scranton and Columbia have solicited the aid of the Pennsylvania State Veterinary Sanitary Board to test the cattle furnishing their milk-supply.

Secretary Edge will ask for one hundred thousand dollars at the next session of the Pennsylvania Legislature, to more thoroughly press the work of eradicating contagious and infectious diseases from the State. Some definite ideas are now being gained as to the prevalence of tuberculosis by the present work of the Board.

State Veterinarian Pearson has been investigating quite an outbreak of rabies in Centre and Union counties.

